# Does variability of footfall kinematics correlate with dynamic stability of the centre of mass during walking?

**DOI:** 10.1371/journal.pone.0217460

**Published:** 2019-05-31

**Authors:** Niklas König Ignasiak, Deepak K. Ravi, Stefan Orter, Seyyed Hamed Hosseini Nasab, William R. Taylor, Navrag B. Singh

**Affiliations:** 1 Institute for Biomechanics, ETH Zürich, Leopold-Ruzicka-Weg 4, Zurich, Switzerland; 2 Department of Physical Therapy, Chapman University, Irvine, California, United States of America; University of Rochester, UNITED STATES

## Abstract

A stable walking pattern is presumably essential to avoid falls. Stability of walking is most accurately determined by the short-term local dynamic stability (maximum Lyapunov exponent) of the body centre of mass. In many studies related to fall risk, however, variability of step width is considered to be indicative of the stability of the centre of mass during walking. However, other footfall parameters, in particular variability of stride time, have also been associated with increased risk for falling. Therefore, the aim of this study was to investigate the association between short-term local dynamic stability of the body centre of mass and different measures of footfall variability. Twenty subjects performed unperturbed walking trials on a treadmill and under increased (addition of 40% body weight) and decreased (harness system) demands to stabilise the body centre of mass. Association between stability of the centre of mass and footfall parameters was established using a structural equation model. Walking with additional body weight lead to greater instability of the centre of mass and increased stride time variability, however had no effect on step width variability. Supported walking in the harness system did not increase centre of mass stability further, however, led to a significant decrease of step width and increase in stride time variability. A structural equation model could only predict 8% of the variance of the centre of mass stability after variability of step width, stride time and stride length were included. A model which included only step width variability as exogenous variable, failed to predict centre of mass stability. Because of the failure to predict centre of mass stability in this study, it appears, that the stability of the centre of mass is controlled by more complex interaction of sagittal and frontal plane temporal and spatial footfall parameters, than those observed by standard variability measures. Anyway, this study does not support the application of step width variability as indicator for medio-lateral stability of the centre of mass during walking.

## Introduction

An efficient walking pattern is characterised by a variety of distinct gait domains, such as pace, rhythm, symmetry, variability and balance [1, 2]. With age, as well as in subjects with neuromotor deficits, all or some of these domains are perturbed, which ultimately results in an increased risk of falling [3–5]. While measures to quantify variability during walking are known to be sensitive in the discrimination of faller and non-faller subjects, their power to estimate fall risk on an individual basis prior to a first fall, remains unclear [3, 6–8]. It is generally assumed that the movement of the centre of mass (CoM) during walking is maintained (returned back to a steady-state after a perturbation) by effectively negotiating the placement of our feet, formally described as the base of support (BoS), and provides the primary means for stabilizing the system [9–12].

In line with this general assumption two different approaches have been considered to evaluate the relationship between movement of CoM and foot placement patterns to maintain stability during locomotion in humans: a) correlating the trajectories of the feet with the movement of CoM (for an exhaustive review on the topic please refer to [12]) in order to *directly* address planned placement of the foot to negotiate balance from one step to the other [11, 13], and b) using step width variability to *indirectly* assess both balance control [1, 7] as well as fall risk [3, 6, 7, 14] during walking over multiple consecutive steps. While a direct approach to establish the relationship between foot placement and the movement of the CoM might seem more valid and physiologically realistic, only individual observations in stroke patients have used this method [15], and hence, such an approach has yet to find clinical uptake, at large. On the other hand, indirect approach of associating step width variability to assess fall risk is well accepted, perhaps due to the widespread availability of easy-to-use technological solutions (e.g. GAITRite system). While there is enough evidence to suggest an association between step-width variability and fall risk, its association with balance control (although assumed) remains largely unexplored.

Assessment of balance control during walking is a seminal issue and a well-researched topic [3, 7, 9, 16–18]. In static situations, the human body behaves like an inverted pendulum such that balance is achieved by maintaining the vertical projection of the CoM within the boundaries of the BoS [19]. As the CoM is in forward motion during walking the simple inverted pendulum model assumptions need to be extended (c.f. Hof [9, 18] for details) in order to incorporate both the projected CoM position as well as the body’s velocity (i.e. the extrapolated centre of mass). Thus, the extrapolated movement of the CoM needs to be maintained within the BoS to achieve balance during dynamic scenarios. As walking is negotiated by alternating the placement of both feet (unilateral stance phases), in order to achieve medio-lateral (ML) stability during walking, the CoM is accelerated medio-laterally throughout the course of walking, such that the extrapolated CoM (XCoM) follows the BoS [9]. Or vice versa, the subsequent steps must be placed according to the position and velocity of the CoM movement in order to maintain stability [13]. By incorporating the velocity of the CoM, Hof’s biomechanical model of stability therefore allows predicting the behaviour of the entire system in the subsequent step. Here, XCoM has extensively been used to investigate the direct relationship between balance control and foot placement, relationship that is *specific* to each step. It remains unknown whether such relationship holds true *iteratively*, i.e. for longer than the immediate (preceding or subsequent) step. Finally, XCoM-based definition of stability or balance control does not provide any indication of system’s ability to respond to perturbations, i.e. might not be well suited to address the dynamic aspects of balance control during walking.

Over longer timespans, dynamic stability has been defined as the ability of a system (here the biomechanical as well as neuromuscular [20]) to respond to small internal or external perturbations [16]. Here, the dynamics of human walking are characterised by aperiodic, fractal-like, chaotic behaviour, thus highlighting temporal dependencies between steps [17, 20–22]. The largest Lyapunov exponent (LyE) is a valid indicator for dynamic stability during walking and thus allows an *overall* quantification of non-linear gait behaviour over the entire duration of an observation [20]. In principle, the LyE measures the maximal rate of spatio-temporal divergence of neighbouring trajectories over a certain time period, after the kinematic gait signal has been transformed into its appropriate state-space. Consequently, a system which is “insensitive to the applied perturbation”, i.e. neighbouring trajectories that have a small rate of divergence or a fast rate of convergence, is interpreted as being dynamically stable [22, 23]. In the context of balance control during walking, it seems entirely plausible that in order to avoid falls, the neuromuscular system aims to stabilise the movement of the CoM during gait. Here variability, evaluated as the standard deviation (or coefficient of variation), of the step-width provides an *overall* assessment of negotiation in foot placement, and thereby and indirect indicator of balance control. Similar to spatial step-width variability in the ML direction, variability of temporal parameters of walking e.g. footfall kinematics, represents the domain of gait rhythmicity, or temporal steadiness in the AP direction [24–27], and such parameters have also been effective in identifying motor deficits and persons with a history of falls [4, 6, 24]. As LyE respects the temporal dynamics of the system, it might therefore be suitable for explaining the empirical relationships between balance control, rhythmicity, and even fall risk. We postulate that the LyE of the CoM movement trajectory in the ML direction over the entire duration of walking is a primary outcome measure to assess the neuromuscular system.

The key issues addressed within this study are: a) while it has been shown that foot placement supports balance control *specific* to each step, it is unknown whether this support is *iterative* (i.e. the role of preceding steps) and b) whether step-width variability is an *overall* indicator of balance control. Resolving these issues as well as investigating whether these associations change with weight-based perturbations will provide deeper understanding for the cross-modal interactions between balance control and the corresponding BoS, in both step *specific* as well as in an *overall* manner (popular among the clinical context). By assessing the relationship between dynamic stability of the CoM as a primary aim of the human neuromuscular control system and the respective footfall kinematics, it might be possible to extend our understanding of the empirical findings of disrupted rhythmicity and balance during walking and the risk of falling.

The aim of this study was to investigate which aspects of footfall kinematics are capable of predicting stability of the CoM, as determined by both XCoM as well as LyE. Here, we investigated the association between the XCoM and temporal and spatial footfall kinematics, not only on a step *specific* manner, but also *iteratively* (over 5 previous steps). Finally, we investigated whether step width variability is an overall assessment of dynamic balance control, by associating it with LyE of CoM. We hypothesize that specific weight-based perturbations will have distinct effects on the stability of the CoM in the ML direction and that those effects are accompanied by quantifiable changes in footfall kinematics over two different time scales, i.e. at a specific step as well as over the entire duration of a walking episode.

## Methods

Prior to study participation each of the 20 healthy subjects (10 males and 10 females) provided written informed consent. The mean (SD) age, height and body mass of the participants was 27.0 (4.2) years, 175.7 (8.9) cm, and 71.6 (10.9) kg respectively. None of the subjects presented any form of musculoskeletal or neurological disorder or pain. The entire study protocol was approved by the local ethics committee (ETH Zurich Ethikkommission) and was conducted in accordance with the Declaration of Helsinki.

In order to experimentally investigate the influence of footfall kinematics on CoM stability, subjects were asked to perform barefoot walking trials with different CoM stabilisation requirements. Each subject walked for at least 5 minutes on a motorized treadmill at their self-selected walking speed (“*normg*” condition; group average: 0.96±0.10m/s). Self-selected speed was determined by subjects starting to walk on the gradually accelerating treadmill until subjects reported to have reached a comfortable walking pace. After a short period of time to accommodate to the selected speed in such a manner, subjects were asked whether the speed was still comfortable and wherever necessary the treadmill speed was increased or decreased. The recording of the trial was started with subjects already walking at their comfortable speeds. At the end of the trial the recording was stopped before the treadmill was halted. Additionally, to the *normg* trial, in order to change the requirements of CoM stabilisation, subjects performed four walking trials with +20% bodyweight (*wei20*) and +40% bodyweight (*wei40*) applied with a weight vest, as well as -20% bodyweight (*har20*) and -40% bodyweight (*har40*) unloading in a clinical harness system. A whole-body marker set, consisting of 62 reflective markers, was used with at least 4 markers on each segment of the body, including: both feet, shanks, thighs, upper arms and forearms, the pelvis, the upper trunk and the head. Kinematic data was collected using an optical motion-capture system (Vicon Motion Systems Ltd, UK) at a sampling frequency of 100Hz.

### CoM kinematics

The 3D position of the CoM for each subject was estimated using an available OpenSim model [28] as follows: Firstly, the model segments were scaled to represent the body dimensions of each subject. Here, an overall scaling factor was assigned to each segment based on the distances between specific bony landmarks. Next, an inverse kinematics approach was applied for the entire 5 min walking trial in order to generate continuous trajectories of the movement of each segment CoM. The mass of each segment was derived as a ratio of the total body mass of a subject, as defined in the OpenSim model. Finally, 3D trajectory of the virtual body CoM (defined as the weighted average of all body segment CoMs) was obtained using the *Body Kinematics tool* in OpenSim (version 3.3).

The trajectory of the estimated body CoM in the ML direction was then used to evaluate each subject’s short-term local dynamic stability (i.e. LyE) during walking. In order to ensure that all time series were of equal lengths, all trials were cropped to the length of the shortest trial in the study, followed by the additional removal of the initial and final 10 seconds of each trial to avoid transients. Afterwards the data was down-sampled to 60Hz, as generally suggested for non-linear analysis of gait dynamics [22]. Prior to performing state-space reconstructions, the appropriate number of embedded dimensions and time lag were identified using the false nearest neighbour and average mutual information approaches respectively for each trial on an individual basis [22]. Subsequently, Wolf’s algorithm was used to estimate the LyE for the trajectory [29] of the whole-body CoM in the ML direction.

### Footfall kinematics

The trajectories of all markers on both feet (heel, base metatarsus three, first–and second metatarsus heads) were used to extract the footfall kinematics. Kinematics were low-pass filtered (Butterworth, fourth-order, 25Hz cut-off frequency), prior to the identification of heel-strike and toe-off events using a foot velocity algorithm [30]. Two consecutive heel strikes of the same leg were defined as a stride. Stride time was calculated for both feet independently as the time elapsed between two ipsilateral consecutive heel-strikes, and stride length was defined as the Euclidean normg vector between the x-y foot coordinates at toe-off and its consecutive heel-strike. Step width and length were calculated in the ML direction and AP direction respectively, as the distance between the line of progression of two consecutive ipsilateral heel strikes and the position of the contralateral heel marker at its heel strike. Finally, variability of footfall parameters was evaluated for each foot separately by calculating the coefficient of variation of the parameters obtained.

#### Extrapolated centre of mass

COM velocity (*COMv*) were computed from the first derivative of the calculated COM position (*COMp*) in 3D. The calculated *COMv* data were low pass filtered with a 4^th^ order bidirectional Butterworth filter with a cut off frequency of 20Hz. Further, extrapolated centre of mass (XCoM) was derived according to the following definition, adapted from [9] and [31].

$$XCoM=COMp+\frac{COMv}{\sqrt{\frac{g}{l}}}$$ (1)

Where ‘*g*’ is the acceleration due to gravity and ‘*l*’ is the pendulum length and is defined as the distance between heel marker (placed over the calcaneus bone) to the COM position at heel strike (average over each weight conditions trial per subject).

### Statistical analyses

#### Associating footfall kinematics with CoM stability specific to each step

In order to summarize the relationship between footfall kinematics and the movement of the CoM multiple linear regression analyses using the enter approach was performed. Here, the independent variables were spatial placement of foot (step width and length evaluated successively from both left and right foot), as well as the first and the second difference in the spatial placement of the foot (difference between the current step width and length to the previous two step widths and lengths). This ensured that not only the current step, but also the contributions of the preceding steps from both limbs were used as predictors. In total 13 independent variables including the intercept were used to predict the dependent variable, XCoM in ML, for each participant and perturbation condition separately, using the *regress* procedure in Matlab. In summary, multiple regression tested the following equation at the significance level of 0.05:

$$\begin{array}{c} {{XCoM\;in\;ML}_{i}|}_{part,cond}=\beta_{0}\ldots \\ +\beta_{1,2}{\left(Step\;width\right)}_{i,limb}+\beta_{3,4}{\left(Step\;length\right)}_{i,limb}\ldots \\ +\beta_{5,6}({\delta_{i-1}^{i}(Step\;width)}_{limb})+\beta_{7,8}(\delta_{i-1}^{i}{\left(Step\;length\right)}_{limb})\ldots \\ +\beta_{9,10}({\delta_{i-2}^{i}(Step\;width)}_{limb})+\beta_{11,12}{\left(\delta_{i-2}^{i}(Step\;length\right)}_{limb}) \end{array}$$ (2)

where, *XCoM*, *step width and step length* were evaluated as described previously, *i* indicates the current heel strike with indices i-1 and i-2 denoting previous gait events, *limb* indicates the heel strike from either the left or the right limb, *δ* indicates the first and the second difference in step width and length from up to two heel strikes, *β*_1…12_ denote regression coefficients for the respective independent variable, while *β*_0_ is the intercept. The multiple regression was run separately for each participant (*part*) and perturbation condition (*cond*).

The coefficient of determination, R^2^, was used to determine the strength of the prediction for each subject and condition individually.

#### Establishing step width variability as an overall assessment of balance control

In order to establish step width variability as an overall assessment of balance control, the effect of the gait perturbations on the parameters: largest lyapunov exponent of the CoM in ML (LyE), coefficient of variation of stride time (CV-ST), and coefficient of variation of step width (CV-SW), was evaluated using a mixed-model repeated measures ANOVA with subjects as random and perturbation conditions (*normg*, *wei20*, *wei40*, *har20*, *har40)* as fixed effects. Post-hoc comparisons were made using the least significant difference procedure. The significance level was set at 5%. The ANOVA was conducted in SPSS (SPSS v25, IBM Corp, United States).

In order to investigate the relationship between individual footfall kinematics and walking balance, a structural equation model (SEM) was established (AMOS v23, IBM Corp, United States). Firstly, the association between the exogenous variables CV-SW and CV-ST with the endogenous variable LyE was investigated with a simple linear regression model. Secondly, a model was established which includes both exogenous variables as well as their dependency. Thirdly, the coefficient of variation of step length (CV-SL) was introduced as additional exogenous variable with the aim to maximise the explained LyE variance. The quality of SEM models can be evaluated using a chi-square statistic. However, in order to assess chi-square statistic, the estimated degrees of freedom need to be smaller than the number of distinct degrees of freedom. In order to reduce the number of degrees of freedom in the developed SEM models, associations between exogenous variables were removed from the model in case these were smaller than 0.1. Finally, two more SEM models were developed. For the SEM, the data of all trials was included after removal of outliers (i.e. Z score > 3), which resulted in a total of 88 cases (of 5 min walks). The data was tested for multivariate normgality, before fitting the models using the maximum likelihood approach within SEM. In order to assess the performance of the developed model, it was compared to a *saturated* (fully explanatory; parameter estimates = degrees of freedom) and *independence* (fully uncorrelated; no relationships between variables) model. Significance was assumed at p<0.05.

## Results

### Associating footfall kinematics with CoM stability specific to each step

**Fig 1:**
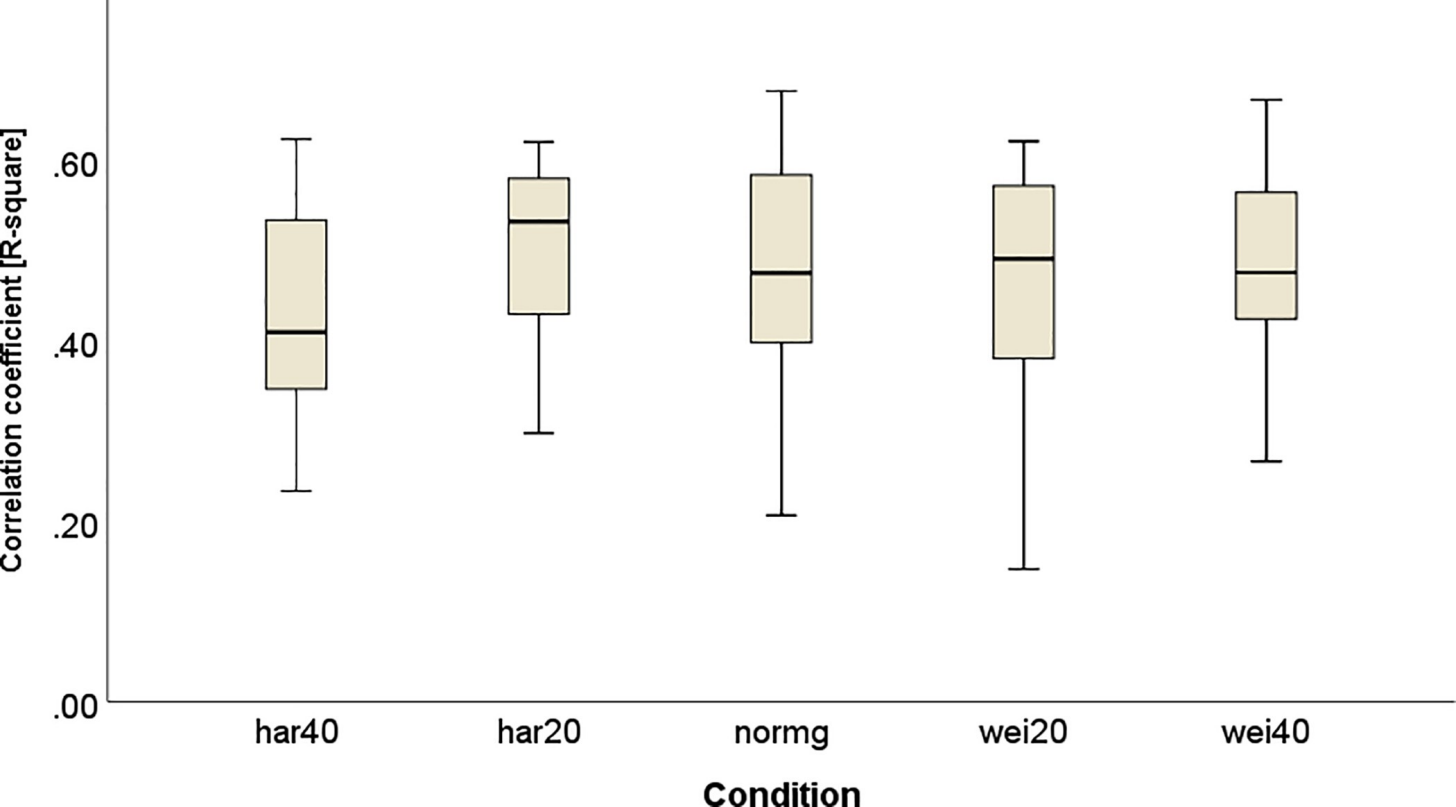
R^2^ values from the multiple linear regression between footfall kinematics and XCoM in the iterative step specific analysis for each experimental condition.

The multiple linear regression analyses revealed that footfall kinematics significantly predicted the movement of the CoM, with R^2^ values ranging from 0.15 in the *wei20* condition to 0.68 in the *normg* condition (Fig 1).

### Establishing step width variability as an overall assessment of balance control

**Fig 2:**
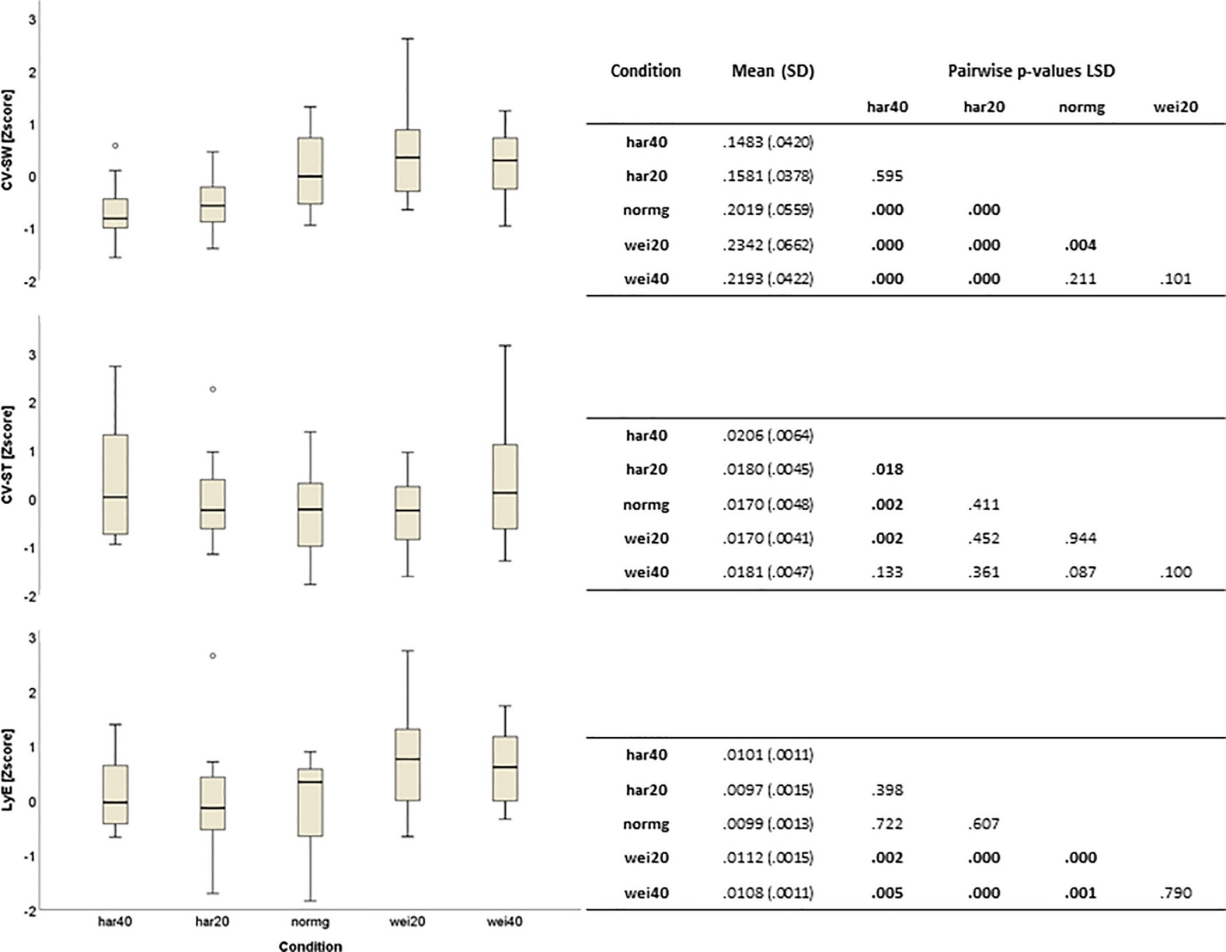
Effect of the walking conditions on the three main outcome parameters: CV-SW, CV-ST and LyE. Mean (SD) in the table are in units of the outcome, for CV-SW and CV-ST in %, for LyE the exponent value. Bold p-values indicate significance at 5% alpha level in the LSD post-hoc test.

The analysis of variances revealed a significant effect of the walking perturbation on LyE (F_(4,95)_ = 7.9; *p*<0.001), CV-ST (F_(4,72)_ = 3.5; p = 0.011) and CV-SW (F_(4,69)_ = 19.1; p<0.001). For LyE, the *wei40* condition resulted in a significant increase in LyE compared to the *normg* (p = 0.001), *har20* (p<0.001) and *har40* (p = 0.005) conditions (Fig 2). CV-ST was significantly increased in the *har40* (p = 0.02) and showed a trend in *wei40* (p = 0.09) compared to the *normg* trials. Walking in the harness system resulted in a significant decrease in CV-SW as compared to *normg* (*har40*: p<0.001; *har20*: p<0.001), *wei20* (*har40*: p<0.001; *har20*: p<0.001) and *wei40* (*har40*: p<0.001; *har20*: p<0.001) conditions (see supplementary material S1 Table).

**Fig 3:**
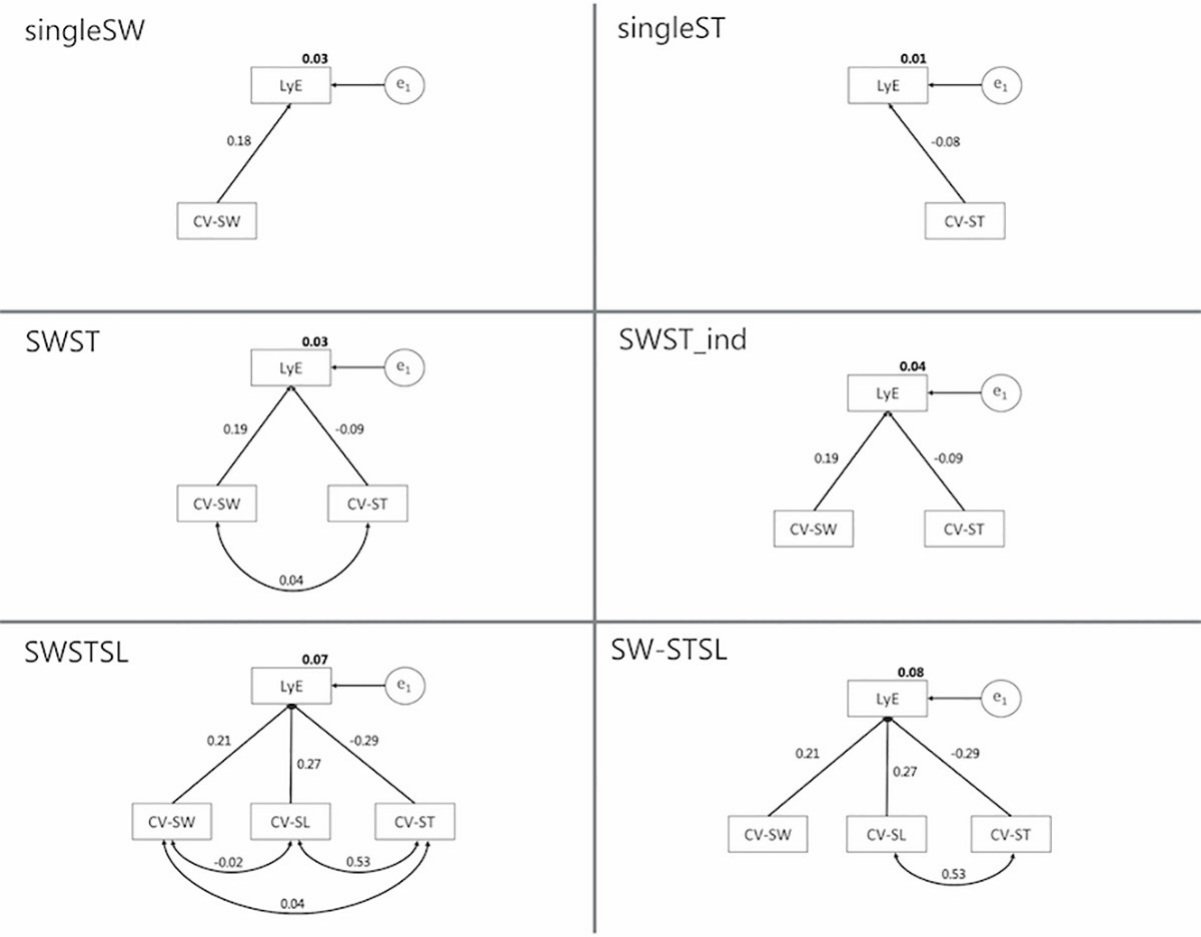
Overview of all SEM models with total variance explained (bold) and standardised correlation coefficients.

CV-SW independently was not correlated to LyE (Fig 3). CV-ST explained 3% of the variance of LyE in the linear regression model (singelST; Table 1). Including both endogenous variables in a multiple regression model (SWST) did not improve the variance explained (r^2^ = 0.03). Since these models were *just identified* (i.e. degrees of freedom (df) = 0), no chi-square statistic could be computed. By removing the association between CV-SW and CV-ST in the SWST_ind model, one df was released. This model explained 4% (p = 0.51) of the variance of LyE, with CV-SW having a stronger association than CV-ST. Inclusion of the parameter CV-SL increased the variance explained in the SWSTSL model to an r^2^ = 0.07, with correlation coefficients of r = -0.29, 0.27 and 0.21 for CV-ST, CV-SL and CV-SW respectively. By removing the small associations (<0.1) between CV-SW and CV-SL, as well as those between CV-SW and CV-ST two degrees of freedom were released. The so developed SW-STSL model explained 8% of the variance of LyE, with a non-significant chi-square test (p = 0.45), indicating that the results of the SW-STSL model are consistent with the experimental data (see supplementary material S1 Table).

**Table 1:** Comparison of SEM models, with tests for multivariate normgality, distinct and estimated degrees of freedom, total variance explained, Chi-square statistic, Root-mean square error, Chi-square goodness of fit and Akaike information criterion.

Model	Normgality (C.R.)	Distinct Df	Estimate Df	Df	r^2^	Χ^2^ probability (p-value)	RMSEA	CMIN/df	AIC	AIC sat.	AIC ind.
singleSW	0.84	3	3	0	0.03	-	-	-	6	6	6.16
singleST	1.09	3	3	0	0.01	-	-	-	6	6	4.56
SWST	0.19	6	6	0	0.03	-	-	-	12	12	9.35
SWST_ind	0.19	6	5	1	0.04	0.51	0.00	0.44	10.44	12	9.35
SWSTSL	1.52	10	10	0	0.07	-	-	-	20	20	80.47
SW-STSL	1.52	10	8	2	0.08	0.45	0.00	0.80	17.59	20	80.47

C.R.: Critical ratio (values > 1.96 indicate significance, with alpha 5%); Df: Degrees of freedom; RMSEA: Root mean square error of approximation; AIC: Akaike information criterion; sat.: Saturated model; ind.: Independent model.

## Discussion

The aim of this study was to investigate the association between footfall kinematics and the stability of the CoM iteratively over a few specific steps as well as over the entire duration of a walking trial. Our findings provide insights on the role of foot placement in stability of the CoM, as well as whether summary spatio-temporal variability (particularly step width variability) measures are indicators of balance control. Interestingly we found that XCoM can be predicted (up to about 68%) using the step width and length from the current and 4 preceding steps. However, variability of step width and stride time independently, but also in combination summarized over an entire walking trial, were not able to predict dynamic stability of the CoM as indicated by LyE. A combination of step width, stride time and step length variability only predicted 8% of CoM stability variance, and hence questions the application of footfall kinematic variability as a surrogate measure for balance control. Finally, our analysis revealed that the association between step-width (and length) and XCoM was lowest in the 40% weight-reduction condition, while the largest LyE was observed for the 40% weight-addition condition.

The stabilising harness condition resulted in a significant decrease in CV-SW, and destabilisation of walking with the additional weight showed an increase in CV-SW in the wei20 condition. Variability of step width is thought to reflect balance performance during walking, since the placement of the feet are essential for the active stabilisation of medio-lateral CoM movements [26, 32]. The harness system provides passive stabilisation of the CoM and generally restricts the movements of the CoM in all directions, thus possibly also explaining the observed reduction in CV-SW [33–35]. The opposite effect, destabilisation of CoM movements, was achieved by the addition of weight, as suggested by the observed elevated levels of LyE. Although our study only revealed an increase in CV-SW with the addition of weight in the wei20 condition, other studies have found significant increases in CV-SW and variability of CoM kinematics while walking with additional weight [36, 37]. Therefore, it appears that stabilising harness systems resulted in reduced medio-lateral variability of the CoM. On the other hand, additional weight led to a significant destabilisation of the CoM in the medio-lateral direction (i.e. LyE in wei20 and wei40), which was not accompanied by a similar increase in CV-SW in the wei40 condition (Fig 2). Similarly, a significant reduction in CV-SW in the harness conditions was not accompanied by a further reduction in LyE. Consequently, no linear association between CV-SW and LyE could be established across the five experimental condition. Hence, an increase (or decrease) in CV-SW during perturbed walking, might be insufficient in predicting the LyE of the CoM.

Our results on the relationship between foot placement and the movement of the CoM, are similar to those reported in the literature [13]. While most of these reports have been proposed for a single step, our neuromuscular system works under stringent temporal constraints (requires approx. 600 ms [38]) when processing sensorimotor signals during walking. Thus, it could be argued that the locomotor adaptation in the event of a destabilising perturbation may therefore happen gradually over more than one single step. Thinking backward, we suspect that the current foot placement may still be an adaptation following a perturbation that happened a while ago [39]. In line with this argument we proposed the analysis of foot placement based on state of 5 previous steps (as presented in Eq 3 going as far back as 2^nd^ difference between both step width and length, but the knowledge of how many steps required for this approach is limited, subject and perturbation -specific and renders separate investigations). Therefore, we contend that, it’s not just the previous state, it’s the set of previous states from which the system at the current state gathers information on stability. Such an *iterative* setup allows flexibility to the neuromuscular system for both planning and execution of foot placement such that stability is maintained. We found support for such an iterative process as our analysis predictions maintained between 15–68%. More importantly these associations seem subject-specific, and were generally not influenced by perturbation conditions, except in 40% weight reduction condition.

The behaviour of variability of stride time in response to the perturbation showed an increase of CV-ST in the *har40* condition compared to the *normg* condition, as well as a trend towards an increase in the *wei40* condition (*normg* vs. *wei40;* p = 0.087). An increase in stride time variability indicates reduced rhythmicity of the gait pattern and it has been shown previously that walking with body weight support increases variability of lower extremity temporal kinematics [24, 25, 27, 40]. In this context, it has been hypothesized that reduced proprioceptive load receptor input might play an important role for the maintenance of a rhythmic gait pattern [35, 40, 41]. Moreover, it has been reported that increasing load on the body during upright standing results in a reduction of somatosensory evoked potentials from mechanoreceptors of the plantar foot [42]. Also, patients with diabetic neuropathy, who typically present reduced mechanoreceptor sensitivity in the lower extremities, are found to have elevated levels of stride time variability [43, 44] and increased fall risk [45, 46]. Therefore, we hypothesize that reduced availability of mechanoreceptive information during decreased as well as increased body weight conditions results in the disturbance of gait rhythmicity as presented in this study. However, there was no clear association between stability of the CoM and gait rhythmicity, since in the harness conditions CV-ST was significantly elevated but not LyE, and conversely that during load carriage LyE was significantly increased, whereas CV-ST only showed a non-significant trend. Again, since there was no linear association between CV-ST and LyE it appears that the empirical association between increased fall risk and decreased gait rhythmicity [3, 6, 14, 24, 45] might not be established via a decrease in CoM stability, but probably via other mechanisms such as an increase in toe clearance variability and an increased risk for stumbling as well as their combination [47].

Most of our SEM models were not capable of associating measures to quantify variability of footfall kinematics with the stability of the CoM as represented by LyE. In particular, the *singleSW* model (i.e. ordinary linear regression model) failed to reveal any correlation between LyE of CoM and CV-SW. In contrast, Young and Dingwell reported that up to 41% of trunk stability can be explained by step width variability [48]. However, there are considerable differences between the two studies, which might explain the inconsistent results. For example, the reported association was found for the stability measure of maximum Floquet multipliers as compared to short-term local stability (i.e. LyE) reported here. Although both measures are thought to quantify the stability of a system in response to perturbations, the former measure assumes strict periodicity as opposed to aperiodicity of the latter, and in general only a weak association has been found between the two measures [17]. Interestingly, the *SW-STSL* SEM model indicated a non-significant difference between the observed and predicted covariance matrices, a good relative CMIN value and a reasonable AIC value, which speaks in favour of the model ability to predict CoM stability. However, the magnitude of the association was too low with only 8% of CoM stability variance explained by *SW-ST-SL* variables, to provide reasonable predictions. It appears that CoM stability is a complex mechanism requiring intricate spatial and temporal control of foot strike patterns. In a similar manner, Dingwell and coworkers showed that changing either step width or stride length patterns results in significant changes in the short-term local stability of the trunk [48]. It appears that a complex interaction of multifaceted footfall mechanisms govern trunk stability. For example, an increase in step width variability was associated with either increased or decreased medio-lateral trunk stability, depending on the average step length. On the other hand, a decrease in stride time variability was associated with either increased or decreased trunk stability, depending on the average step width [48]. Similarly, in this study, a decrease in CV-SW seemed to occur independently of a change in CoM stability, but decreased CoM stability was associated with increased step width variability. In a similar manner, an increased CV-ST might occur with or without a change of LyE. These observations therefore highlight the biomechanical and neuro-motor control complexity that is required for maintaining dynamic stability during walking. Likewise, these observations emphasize the need for similarly holistic explanatory models that allow inclusion of several gait parameters to predict walking stability, and thereby justify the application of tools such as structural equation modelling in biomechanical research.

A general limitation of many statistical tools to investigate the relationships between sets of variables is the assumption of linearity, which is also the case in the model applied here. Applicability of the SEM model might therefore be limited by the non-linear behaviour of CV-SW, CV-ST and LyE in respect to walking conditions. Rather than including all conditions on one model, a separate analysis of walking conditions might have been more appropriate, but this would have required a much larger sample size. Also, it should be considered that gait measures might underlie floor effects when being investigated in a high performing group, such that a further change in gait performance could be observed with these metrics (i.e. due to the highly stable walking patterns in the *normg* condition, no additional stability was observed in the harness trials). Therefore, the rather low sample size as well as the fact that a healthy young group was investigated here should be considered when drawing conclusions regarding the relationship between dynamic walking stability and variability of footfall kinematics reported in this study.

In the young and healthy group investigated in this study, additional weight led to decreased dynamic stability during walking on a treadmill. A stabilising harness system did not provide further stability to those subjects. Stride time variability increased when walking with additional weight, and step width variability reduced during walking in the harness system. During the iterative step-by-step observation, footfall kinematics explain about 50% of the variation in the centre of mass trajectory. However, a multiple step observation and the observation of footfall variability over an entire walking trial failed and those two footfall parameters were not able to predict dynamic stability in the medio-lateral direction in a structural equation model. The best model, after additional inclusion of stride length variability, allowed to significantly explain only 8% of the variance found in dynamic stability. It appears that dynamic stability of the centre of mass requires the complex control of spatial and temporal gait parameters in the frontal as well as the sagittal plane, but is not sufficiently described by the variability of step width alone. Furthermore, it appears that the unordered observation of footfall placement (i.e. variability measures of step width and stride time over one trial) removes the well-established step-by-step association between foot placement and CoM stability in clinical multiple steps including observations. Thus, dynamic stability of the walking pattern cannot be assessed by only evaluating step width variability. Consequently, the claim of step width variability being representative of dynamic walking balance should be revised.

## Supporting information

S1 Table The excel table includes data for each participant each condition for the main outcome parameters CV-SW, CV-ST, CV-SL and LyE in both nominal (Worksheet Data_ANOVA) and standardized (z-scores Worksheet Data_SEM). (XLSX)
